# New Sinularianin Sesquiterpenes from Soft Coral *Sinularia* sp.

**DOI:** 10.3390/md11124741

**Published:** 2013-12-02

**Authors:** Bin Yang, Shengrong Liao, Xiuping Lin, Junfeng Wang, Juan Liu, Xuefeng Zhou, Xianwen Yang, Yonghong Liu

**Affiliations:** CAS Key Laboratory of Tropical Marine Bio-resources and Ecology/Guangdong Key Laboratory of Marine Materia Medica/Research Center for Marine Microbes, South China Sea Institute of Oceanology, Chinese Academy of Sciences, Guangzhou 510301, China; E-Mails: bingo525@163.com (B.Y.); ljrss@126.com (S.L.); xiupinglin@hotmail.com (X.L.); junfeng1982a@163.com (J.W.); ljuan2010@qq.com (J.L.); xfzhou@scsio.ac.cn (X.Z.); xwyang@scsio.ac.cn (X.Y.)

**Keywords:** soft coral, *Sinularia* sp., sesquiterpenes, sinularianins, NF-κB

## Abstract

Four new sesquiterpenes, sinularianins C–F (**3**–**6**), together with known sinularianins A (**1**) and B (**2**) were identified from a South China Sea soft coral *Sinularia* sp. Compounds **1**–**6** were evaluated for inhibition of NF-κB activation using the cell-based HEK293 NF-κB luciferase reporter gene assay. Compounds **1** and **4** were exhibited a potent effect with inhibitory rates of 41.3% and 43.0% at the concentration of 10 µg/mL, respectively.

## 1. Introduction

The genus *Sinularia* is the most widely distributed soft coral, consisting of almost 90 species, of which more than 50 have been chemically examined [1,2,3,4,5]. Up to now, *Sinularia* has yielded many metabolites, including sesquiterpenes, diterpenes, alkaloids, and polyhydroxylated steroids [6,7,8,9,10,11,12]. These metabolites display a wide range of biological activities, such as antimicrobial, anti-inflammatory, and cytotoxic activities [13,14,15,16,17,18]. In our endeavor to explore the bioactive secondary metabolites from marine invertebrates, sinularianins A (**1**) and B (**2**) were reisolated along with four new sesquiterpenes, sinularianins C–F (**3**–**6**) from soft coral *Sinularia* sp., collected at Dongluo Island, Hainan province, China, at a depth of 10 m. Sinularianin A and B have been isolated from the Formosan coral *Sinularia* sp., but their anti-inflammatory activation were tested for the first time. Similar sesquiterpenes had been isolated mostly from the plant *Valeriana officinalis*, which was used as an anti-inflammatory remedy in Europe, and were active as inhibitors of NF-κB [19]. In this paper, we describe the isolation, structure elucidation, and the NF-κB inhibitory potential of these compounds.

## 2. Results and Discussion

**Figure 1:**
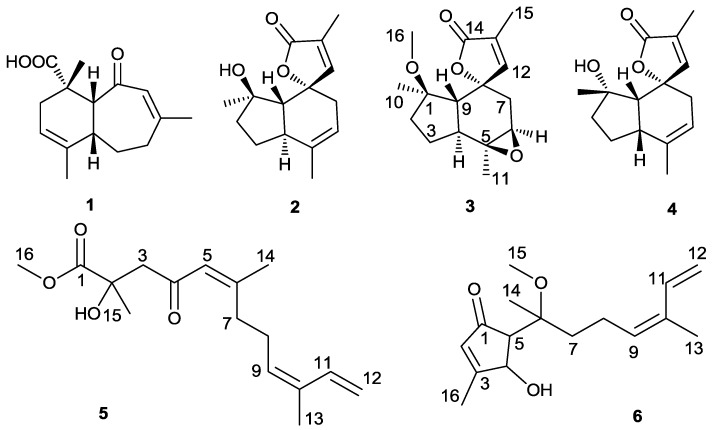
Structures of the compounds **1**–**6**.

The soft coral *Sinularia* sp. was dissolved in 85% EtOH, and the extract separated by silica gel column chromatography, Sephadex LH-20, and semi-preparative HPLC to obtain new sesquiterpenes, sinularianins C–F (**3**–**6**), and two known compounds (**1**, **2**) (Figure 1).

Sinularianins A (**1**) and B (**2**) were previously isolated from the soft coral *Sinularia* sp., collected off the northeastern Taiwan coast, in May 2004, at a depth of 10 m. Sinularianin A (**1**) possesses an unprecedented bicyclic skeleton sinulariolane. Sinularinin B (**2**) was the only example of valerenane-related sesquiterpene with a spiro-butenolide moiety [10]. The valerenane-related sesquiterpenes had been firstly reported from the plant *Valeriana officinalis* [20,21], and several representatives have been reported from a marine alga [22] and a soft coral [23]. Sinularinin A (**1**) and B (**2**), were reisolated and identified by comparison of their ^1^H and ^13^C NMR data with those reported [10].

**Figure 2:**
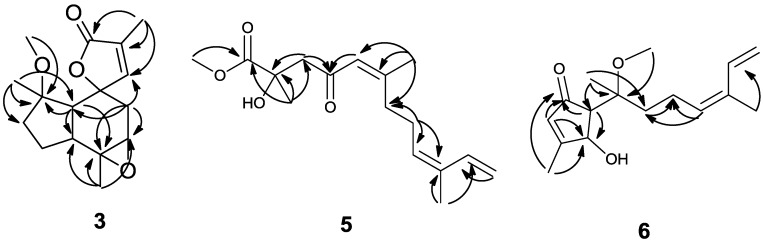
Selected HMBC correlations (H → C)of compounds **3**, **5**, and **6**.

Sinularianin C (**3**) was isolated as a colorless oil. Its molecular formula was established as C_16_H_22_O_4_ on the basis of the positive HRESIMS at *m*/*z* 301.1416 (Calcd for C_16_H_22_NaO_4_, 301.1416), indicating six degrees of unsaturation (Supplementary Figure S1). The ^1^H NMR spectrum (Table 1) revealed the presence of four singlet methyls (δ_H_ 1.00, 1.41, 1.86, 3.17), three methylene signals (δ_H_ 1.95, 1H, m; 1.53, 1H, m; 1.96, 1H, m; 1.56, 1H, m; 2.26, 1H, d, *J* = 16.0 Hz; 1.76, 1H, dd, *J* = 16.0, 5.0 Hz), three methine signals (2.00, 1H, d, *J* = 13.0 Hz; 2.43, 1H, m; 3.11, 1H, d, *J* = 5.0 Hz), and one olefinic proton (δ_H_ 7.17, 1H, d, *J* = 1.5 Hz) (Supplementary Figure S2). The ^13^C NMR spectra, together with HSQC, showed 16 signals for four methyls (δ_C_ 10.3, 20.5, 21.3, 50.8), three sp^3^ methylenes (δ_C_ 23.6, 35.7, 38.7), three sp^3^ methines (δ_C_ 41.1, 50.4, 59.5), three sp^3^ oxygenated quaternary carbons (δ_C_ 61.8, 84.2, 86.5), one sp^2^ methine (δ_C_ 154.7), one sp^2^ quaternary carbon (δ_C_ 129.5), and one carbonyl carbon (δ_C_ 175.6) (Supplementary Figures S3 and S4). Both the ^1^H and ^13^C NMR spectra of **3** showed a close similarity to those of **2** [10]. However, the close comparison of the ^13^C NMR spectroscopic data of **2** and **3** revealed some differences: one trisubstituted double bond in **2** was changed to the epoxy three-menbered ring (δ_C_ 61.8, 59.5) in **3**, and an additional methoxyl (δ_C_ 50.8, δ_H_ 3.17, 3H, s, H-16) was observed in **3**. This assumption was supported by the correlation of H-11 to C-4, C-5, and C-6, H-6 to C-5, and C-7, H-7 to C-5 and C-6 in the HMBC spectrum (Figure 2). Furthermore, the methoxyl substituent was determined to be connected to position C-1 on the basis of the HMBC correlation from 16-OMe to C-1 (Supplementary Figure S5). 

**Table 1:** ^1^H and ^13^CNMR spectroscopic data for compounds **3** (500/125 MHz, in MeOD, δ in ppm, *J* in Hz) and **4** (in CDCl_3_).

Position	3		4	
	^1^H	^13^C	^1^H	^13^C
1		84.2		78.4
2	1.95 m	35.7	1.92 m	43.1
	1.53 m		1.79 m	
3	1.96 m	23.6	2.01 m	25.9
	1.56 m			
4	2.43 m	41.1	2.90 m	41.8
5		61.8		137.5
6	3.11 d (5.0)	59.5	5.26 s	117.2
7	2.26 d (16.0)	38.7	2.53 m	39.4
	1.76 dd (16.0, 5.0)		1.91 m	
8		86.5		85.6
9	2.00 d (13.0)	50.4	1.55 d (12.5)	55.5
10	1.00 s	21.3	1.26 s	28.5
11	1.41 s	20.5	1.74 s	20.5
12	7.17 d (1.5)	154.7	7.03 d (1.5)	150.9
13		129.5		129.7
14		175.6		174
15	1.86 s	10.3	1.94 d (1.5)	10.6
16	3.17 s	50.8		

**Figure 3:**
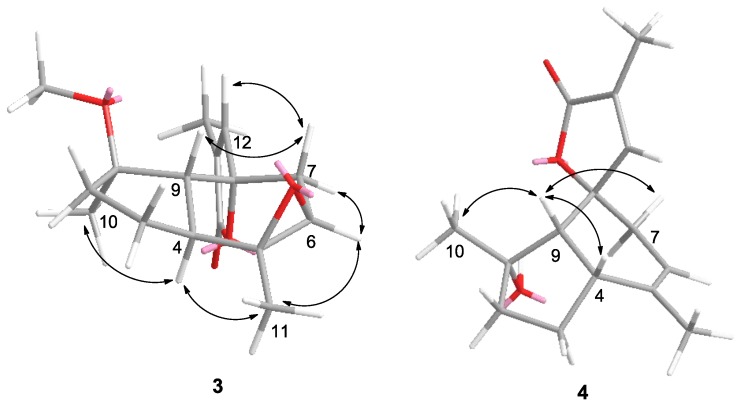
Selected NOE correlations of compounds **3** and **4**.

The relative stereochemistry of **3** was established by the detailed analysis of correlations observed in the NOESY spectrum (Figure 3). In the NOESY spectrum, H-9 showed correlation with H-7β (δ_H_ 2.26, d, *J* = 16.0 Hz), which in turn correlated with H-12, suggesting the β orientations of H-9 and H-12. Furthermore, NOE interactions were observed between H_3_-10/H-4, H_3_-11/H-4, H_3_-11/H-6, and H-6/H-7α (δ_H_ 1.76, dd, *J* = 16.0, 5.0 Hz), while both H_3_-10 and H-4 did not show correlations with H-9, suggesting the *α* orientation of H_3_-10, H_3_-11, H-4, and H-6 (Supplementary Figure S6). 

Sinularianin D (**4**) was isolated as a colorless oil. The ESI-MS showed the [M + Na]^+^ ion at *m*/*z*: 271 (Supplementary Figure S7). Its ^1^H and ^13^C NMR spectroscopic data were also very similar to those of **2** (Supplementary Figures S8 and S9). However, a close inspection of their ^1^H NMR spectroscopic data revealed some difference: H-4 and H_3_-10 were shifted downfield from 2.57 to 2.90, and from 1.12 to 1.26 respectively, and H-9 was shifted upfield from 1.99 to 1.55. This suggested that the configuration at H-1 and H-4 in **4** should be β orientation compared to α orientation in **2**, which was supported by the NOESY experiment (Figure 3). In the NOESY spectrum, H-9 showed correlation with H_3_-10, H-4, and H-7β, suggesting the β orientations of H-4, H-9, H-7β, and H_3_-10 (Supplementary Figure S10). 

Sinularianin E (**5**) was isolated as a colorless oil, and assigned the molecular formula of C_16_H_24_O_4_ by the positive HRESIMS at *m/z* 303.1563 (Calcd for C_16_H_24_NaO_4_, 303.1572) (Supplementary Figure S11). The ^1^H and ^13^C NMR spectroscopic data of **5** indicated sixteen carbon signals: four singlet methyls, four methylenes, three olefinic methines, and five quaternary carbons (Supplementary Figures S12–S14). The ^1^H NMR spectrum showed signals of four olefinic protons (δ_H_ 5.42, 1H, t, *J* = 7.0 Hz; 6.36, 1H, dd, *J* = 17.5, 10.5 Hz; 5.13, 1H, d, *J* = 17.5 Hz; 4.96, 1H, d, *J* = 10.5 Hz), one methoxy group (δ_H_ 3.72), two vinyl methyls (δ_H_ 2.14, s; 1.74, s), and one other methyl (δ_H_ 1.40, s) (Table 2). The gross structure of **5** was established by the assistance of extensive 2D NMR analysis (Figure 2). The methoxycarbonyl was confirmed by HMBC correlations from 16-OMe to C-1. The methyl protons resonating at δ_H_ 1.40 and the quaternary carbon resonating at δ_C_ 72.9 indicated that this methyl and a hydroxyl group should be positioned at C-2 by the HMBC correlations from H-15 to C-1, C-2, and C-3 (Supplementary Figure S15). The olefinic methyls (δ_H_ 2.14, s; 1.74, s) attached at C-6 and C-10 were confirmed by the HMBC correlations from H-14 to C-5, C-6, and C-7 and H-13 to C-9, C-10, and C-11. Furthermore, the HMBC correlations from H-9 to C-8, and C-10, H-12 to C-10, and C-11 established the terminal diene unit. Other key informative HMBC correlations from H-3 to C-2, and C-4, H-5 to C-4, H-8 to C-7, C-9, and C-10, established the planar structure of **5**. The double bond at C-5 was assigned the *Z*-geometry on the basis of the downfield chemical shifts of C-14 (δ_H_ 19.7) [24]. The geometry of the disubstituted double bond (C-9) was determined to be *E* by comparison of the spectral data with those reported in literature [24], whereas the configurations at C-2 remained to be determined. On the basis of above evidences, compound **5** was then identified, and named sinularianin E.

**Table 2:** ^1^H and ^13^C NMR spectroscopic data for compounds **5** and **6** (500/125 MHz, in CDCl_3_, δ in ppm, *J* in Hz).

Position	5		6	
	^1^H	^13^C	^1^H	^13^C
1		176.5		203.2
2		72.9	5.86 s	131.2
3	3.15 d (17.5)	52.9		174.9
	2.80 d (17.5)			
4		199.3	4.79 s	75.3
5	6.01 s	122.9	2.65 s	61.5
6		160.3		78
7	2.22 m	40.9	1.82 m	34.1
			2.12 m	
8	2.34 m	25.8	2.19 m	22.1
	2.20 m			
9	5.42 t (7.0)	130.8	5.52 m	132.3
10		135		134.1
11	6.36 dd (17.5, 10.5)	141	6.36 dd (17.0, 10.5)	141.5
12	5.13 d (17.5)	111.3	5.10 d (17.0)	110.7
	4.96 d (10.5)		4.94 d (10.5)	
13	1.74 s	11.7	1.76 s	11.6
14	2.14 s	19.7	1.03 s	22.7
15	1.40 s	26.2	3.21 s	48.5
16	3.72 s	52.7	2.16 s	15.6

Sinularianin F (**6**) was isolated as a colorless oil. It was assigned a molecular formula of C_16_H_24_O_3_ by positive HR-ESI-MS at *m/z* 287.1613 (Calcd for C_16_H_24_NaO_3_, 287.1623) (Supplementary Figure S16). Analysis of ^1^H and ^13^C NMR data revealed the presence of four methyl groups, three methylene carbons, five methine carbons, and four quaternary carbons (Supplementary Figures S17–S19). The ^1^H NMR spectrum showed signals of five olefinic protons (δ_H_ 5.86, s; 5.52, m; 6.36, dd, *J* = 17.0, 10.5 Hz; 5.10, d, *J* = 17.0 Hz; 4.94, d, *J* = 10.5 Hz), one oxygenated methane (δ_H_ 4.79, s), one methoxy group (δ_H_ 3.21, s), two vinyl methyls (δ_H_ 1.76, s; 2.16, s), and one other methyl (δ_H_ 1.03, s) (Table 2). The HMBC correlations from H-9 to C-8, and C-10, H-12 to C-10, and C-11, H-13 to C-10, and C-11 established the terminal diene unit (Supplementary Figure S20). The key HMBC correlations of H_3_-16 to C-2, C-3, and C-4 and H-2 to C-1, C-3, C-4, and C-5 indicated the presence of a five-membered carbocycle containing a ketone carbonyl and a trisubstituted double bond (Figure 2), as well as by comparison of the data with that of in agreement with the data of cycloabiesesquine A [25]. The two fragments may be connected *via* the correlations of H-15 to C-5, C-6, and C-7, H-14 to C-6 and H-7 to C-6, C-7, and C-8 in the HMBC spectrum. Two double bonds in the molecule possessed 2*Z* and 9*E* configuration on the basis of the chemical shifts of C-16 and C-13 (δ 15.6 and 11.6, respectively) [24,25].

**Scheme 1:**
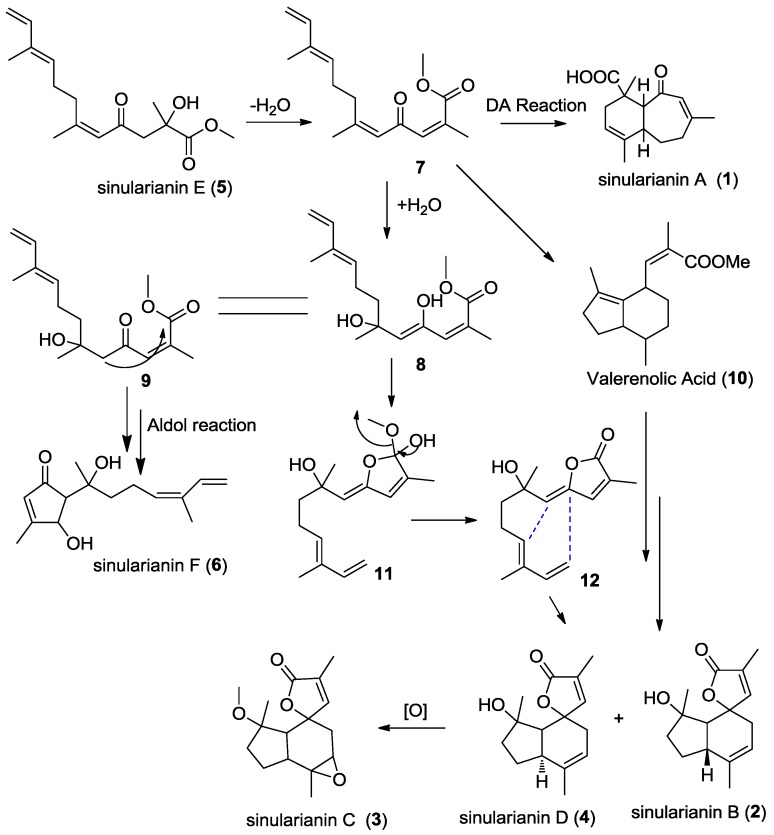
Proposed biosynthetic pathway for **1**–**6**.

Although sinularianins E (**5**) and F (**6**) formally displayed a quite different skeleton from that of sinularianins A–D (**1**–**4**), however, they are actually related to each other. From a biosynthetic aspect, sinularinins A–D (**1**–**4**), and F (**6**) could be generated from sinularinin E (**5**), via different reaction cascades as illustrated in the hypothetical biosynthetic pathway (Scheme 1). As a precursor, sinularianin E (**5**) potentially could be transformed into the key intermediate A by dehydration reaction. Intermediate A could be through different intramolecular Diels Alder cyclization reaction to form sinularianin A (**1**) or valerenolic acid, respectively, and the latter was further modified to produce sinularianin B (**2**). Intermediate A could be also adapted by Michael addition under the H_2_O attack and then immediately lactonized, followed by a DA cyclization to yield sinularianin B (**2**), which after epoxidation and dehydration potentially could be produce epoxide sinularianin C (**3**). Intermediate A might form sinularianin F (**6**) by an aldol condensation.

Nuclear factor-κ B (NF-κB) plays a key role in regulating the immune response to infection. Incorrect regulation of NF-κB has been linked to cancer, inflammatory and autoimmune diseases, septic shock, viral infection, and improper immune development [26]. Compounds **1**–**6** were evaluated for inhibition of NF-κB activation using the cell-based HEK293 NF-κB luciferase reporter gene assay. At concentration of 10 µg/mL, sinularianin A and D exhibits a potent effect with inhibitory rates of 41.3%, and 43.0%, respectively. At the same concentration, other compounds showed moderate effects at the same (Table 3). The past studies have provided biochemical evidence of valerenane-related sesquiterpenes as anti-inflammatory agents acting via the NF-κB inhibitory potential. The valerenic acid (**3**) reduced NF-κB activity to 25% at concentration of 100 µg/mL [19].

**Table 3:** Inhibitory rates of NF-κB activation of compounds **1**–**6**.

Concentration	IR (%)					
	1	2	3	4	5	6
10 µg/mL	41.3	29.6	24.3	43.0	30.0	36.1

## 3. Experimental Section

### 3.1. General Experimental Procedures

The NMR spectra were recorded on a Bruker AC 500 NMR spectrometer with TMS as an internal standard. IR spectra were recorded on a Nicolet 6700 FT-IR spectrometer. UV spectra were recorded on a Shimadzu UV-2600 UV-Vis spectrophotometer. Optical rotations were measured on a PerKin Elmer 341 polarimeter using a 1 dm path length cell. HR-ESI-MS data were measured on AQUITY UPLC/Q-TOF mass spectrometer. ESI-MS data were measured on Bruker’s amaZon SL ion trap LC/MS. Materials for column chromatography were silica gel (100–200, 200–300 mesh, Qingdao Marine Chemical Factory, Qingdao, China), Sephadex LH20 (40–70 µm, Amersham Pharmacia Biotech AB, Uppsala, Sweden), and YMC Gel ODS-A (12 nm, S-50 µm YMC, MA, USA). The silica gel GF_254_ (0.4–0.5 mm) used for TLC were supplied by the Qingdao Marine Chemical Factory, Qingdao, China. HPLC was carried on shimadzu LC-10ATvp with YMC ODS SERIES (YMC-Pack ODS-A, 250 × 10 mm I.D., S-5 µm, 12 nm).

### 3.2. Animal Material

The soft coral *Sinularia* sp. was collected from Dongluo Island, Hainan province of China in July 2009 (7–10 m depth) and identified by Professor Hui Huang, South China Sea Institute of Oceanology, Chinese Academy of Sciences. A voucher specimen (No. 0907010) was deposited in the CAS Key Laboratory of Tropical Marine Bio-resources and Ecology, South China Sea Institute of Oceanology, Chinese Academy of Sciences, Guangzhou, China.

### 3.3. Extraction and Isolation

The fresh soft coral (wet, 6 kg) was extracted three times with 95% EtOH (20 L). The extract was concentrated under reduced pressure, and partitioned between H_2_O (4 L) and CHCl_3_ (4 L); the CHCl_3_ layer (120 g) was further partitioned between 85% EtOH (4 L) and petroleum ether (PE; 4 L) to yield 85% EtOH (34 g) and PE (75.6 g) fractions. The 85% EtOH fraction was separated by silica gel column using CHCl_3_/MeOH to yield 11 portions (Frs. s1–s11). Fr. s3 was purified by silica gel column to yield 12 portions, and portion 10 was further purified with semi-preparative HPLC, eluting with MeOH/H_2_O = 65:35 at a flow rate of 2 mL/min, to afford **1** (6.0 mg) and **2** (7.2 mg). Fr. s5 was purified by Sephadex LH-20 using CHCl_3_/MeOH = 1:1 to yield 3 portions, and portion 1 was further purified with semi-preparative HPLC, eluting with MeOH/H_2_O = 57:43 at a flow rate of 2 mL/min, to afford **5** (2.2 mg) and **6** (2.6 mg). Fr. s6 was separated by silica gel column using PE/EtOAc to yield 7 portions, and portion 1 was further purified with semi-preparative HPLC, eluting with MeOH/H_2_O = 70:30 at a flow rate of 2 mL/min, to afford **3** (2.2 mg) and **4** (3.7 mg). 

Sinularianin C (**3**): Colorless oil; ^1^H- and ^13^C-NMR (see Table 1); HR-ESI-MS *m/z* 301.1416 [M + Na]^+^, (Calcd for C_16_H_22_NaO_4_, 301.1416). 

Sinularianin D (**4**): Colorless oil; 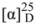 = −6.0 (*c* = 0.01, MeOH); UV (MeOH): λ_max_ (log ε) = 204.2 (1.70); IR (KBr) ν_max_ 3421, 2927, 2854, 1735, 1666 cm^–^^1^ (Supplementary Figure S21); ^1^H- and ^13^C-NMR (see Table 1); ESI-MS *m/z* 271 [M + Na]^+^, 519 [2M + Na]+. 

Sinularianin E (**5**): Colorless oil; ^1^H- and ^13^C-NMR (see Table 2); HR-ESI-MS *m/z* 303.1563 [M + Na]^+^, (Calcd for C_16_H_24_NaO_4_, 303.1572). 

Sinularianin F (**6**): Colorless oil; ^1^H- and ^13^C-NMR (see Table 2); HR-ESI-MS *m/z* 287.1613 [M + Na]^+^, (Calcd for C_16_H_24_NaO_3_, 287.1623).

### 3.4. The Cell-Based HEK293 NF-κB Luciferase Reporter Gene Assay

All compounds were evaluated for inbibition of NF-κB activation using the cell-based HEK 293 NF-κB luciferase reporter gene assay according to the previously reported procedures [19]. 

## 4. Conclusions

The investigation of bioactive natural products from a Hainan soft coral, *Sinularia* sp., has led to the isolation of four new sesquiterpenes, sinularianins C–F (**3**–**6**), along with two other analogues, sinularianins A (**1**) and B (**2**). Compounds **1** and **4** were exhibited a potent inhibitory effect with inhibitory rates of 41.3% and 43.0% at the concentration of 10 µg/mL, respectively. The discovery of new compounds **3**–**6** has added to an extremely diverse and complex array of soft coral sesquiterpenes.

## Supplementary Files

Supplementary File 1 Supplementary Information (PDF, 1507 KB)

## References

[1] Yang B., Zhou X.F., Huang H., Yang X.W., Liu J., Lin X.P., Li X.B., Peng Y., Liu Y.H. (2012). New cembrane diterpenoids from a Hainan soft coral *Sinularia* sp. Mar. Drugs.

[2] Chao C.H., Chou K.J., Huang C.Y., Wen Z.H., Hsu C.H., Wu Y.C., Dai C.F., Sheu J.H. (2012). Steroids from the soft coral *Sinularia crassa*. Mar. Drugs.

[3] Cheng S.Y., Huang K.J., Wang S.K., Duh C.Y. (2011). Capilloquinol: A novel farnesyl quinol from the Dongsha atoll soft coral *Sinularia capillosa*. Mar. Drugs.

[4] Li R., Shao C.L., Qi X., Li X.B., Li J., Sun L.L., Wang C.Y. (2012). Polyoxygenated sterols from the South China Sea soft coral *Sinularia* sp. Mar. Drugs.

[5] Kamel H.N., Slattery M. (2005). Terpenoids of *Sinularia*: Chemistry and biomedical applications. Pharm. Biol..

[6] Su J.H., Wen Z.H. (2011). Bioactive cembrane-based diterpenoids from the soft coral *Sinularia triangular*. Mar. Drugs.

[7] Tsai T.C., Wu Y.J., Su J.H., Lin W.T., Lin Y.S. (2013). A new spatane diterpenoid from the cultured soft coral *Sinularia leptoclados*. Mar. Drugs.

[8] Tseng Y.J., Shen K.P., Lin H.L., Huang C.Y., Dai C.F., Sheu J.H. (2012). Lochmolins A–G, new sesquiterpenoids from the soft coral *Sinularia lochmodes*. Mar. Drugs.

[9] Lai D.W., Li Y.X., Xu M.J., Deng Z.W., van Ofwegen L., Qian P.Y., Proksch P., Lin W.H. (2011). Sinulariols A–S, 19-oxygenated cembranoids from the Chinese soft coral *Sinularia rigida*. Tetrahedron.

[10] Chao C.H., Hsieh C.H., Chen S.P., Lu C.K., Dai C.F., Sheu J.H. (2006). Sinularianins A and B, novel sesquiterpenoids from the Formosan soft coral *Sinularia* sp. Tetrahedron Lett..

[11] Lu M.C., Lee N.L., Tseng S.W., Su J.H. (2011). Sinutriangulin A, a novel diterpenoid from the soft coral *Sinularia triangula*. Tetrahedron Lett..

[12] Putra M.Y., Ianaro A., Panza E., Bavestrello G., Cerrano C., Fattorusso E., Taglialatela-Scafati O. (2012). Sinulasulfoxide and sinulasulfone, sulfur-containing alkaloids from the Indonesian soft coral *Sinularia* sp. Tetrahedron Lett..

[13] Yamashita T., Nakao Y., Matsunaga S., Oikawa T., Imahara Y., Fusetani N. (2009). A new antiangiogenic C-24 oxylipin from the soft coral *Sinularia numerosa*. Bioorg. Med. Chem..

[14] Chai M.C., Wang S.K., Dai C.F., Duh C.Y. (2000). A cytotoxic lobane diterpene from the Formosan soft coral *Sinularia inelegans*. J. Nat. Prod..

[15] Sheu J.H., Chang K.C., Duh C.Y. (2000). A cytotoxic 5α,8α-epidioxysterol from a soft coral *Sinularia* species. J. Nat. Prod..

[16] Chao C.H., Chou K.J., Huang C.Y., Wen Z.H., Hsu C.H., Wu Y.C., Dai C.F., Sheu J.H. (2011). Bioactive cembranoids from the soft coral *Sinularia crassa*. Mar. Drugs.

[17] Shi H.Y., Yu S.J., Liu D., van Ofwegen L., Proksch P., Lin W.H. (2012). Sinularones A–I, new cyclopentenone and butenolide derivatives from a marine soft coral *Sinularia* sp. and their antifouling activity. Mar. Drugs.

[18] Wright A.D., Nielson J.L., Tapiolas D.M., Liptrot C.H., Motti C.A. (2012). A great barrier reef *Sinularia* sp. yields two new cytotoxic diterpenes. Mar. Drugs.

[19] Jacobo-Herrera N.J., Vartiainen N., Bremner P., Gibbons S., Koistinaho J., Heinrich M. (2006). NF-κB modulators from *Valeriana officinalis*. Phytother. Res..

[20] Bos R., Hendriks H., Bruins A.P., Kloosterman J., Sipma G. (1986). Isolation and identification of valerenane sesquiterpenoids from *Valeriana officinalis*. Phytochemistry.

[21] Birnbaum G.I., Findlay J.A., Krepinsky J.J. (1978). Stereochemistry of valerenane sesquiterpenoids-crystal-structure of valerenolic acid. J. Org. Chem..

[22] Mao S.C., Guo Y.W., Shen X. (2006). Two novel aromatic valerenane-type sesquiterpenes from the Chinese green alga *Caulerpa taxifolia*. Bioorg. Med. Chem. Lett..

[23] Kobayashi M., Yasuzawa T., Kyogoku Y., Kido M., Kitagawa I. (1982). Three new ent-valerenane sesquiterpenes from an Okinawan soft coral. Chem. Pharm. Bull..

[24] Bowden B.F., Coll J.C., Desilva E.D., Decosta M.S.L., Djura P.J., Mahendran M., Tapiolas D.M. (1983). Studies of Australian soft corals. XXXI. Novel furanosesquiterpenes from several *Sinularian* soft corals (Coelenterata, Octocorallia, Alcyonacea). Aust. J. Chem..

[25] Yang X.W., Ding Y.Q., Li X.C., Ferreira D., Shen Y.H., Li S.M., Wang N., Zhang W.D. (2009). Cycloabiesesquine A, a unique sesquiterpenoid from *Abies delavayi*. Chem. Commun..

[26] Peddibhotla S., Shi R.X., Khan P., Smith L.H., Mangravita-Novo A., Vicchiarelli M., Su Y., Okolotowicz K.J., Cashman J.R., Reed J.C. (2010). Inhibition of protein kinase C-driven nuclear factor-kappa B activation: Synthesis, structure-activity relationship, and pharmacological profiling of pathway specific benzimidazole probe molecules. J. Med. Chem..

