# Supplementary material for: New Sinularianin Sesquiterpenes from Soft Coral Sinularia sp
Source: Mar Drugs. 2013 Dec 2;11(12):4741–50. doi: 10.3390/md11124741 (PMC3877883; doi:10.3390/md11124741)
Supplement: Supplementary File 1 — Supplementary Information (PDF, 1507 KB) [file marinedrugs-11-04741-s001.pdf]

Supplementary Information

Figure S1. HR-ESI-MS of sinularianin C (**3**).

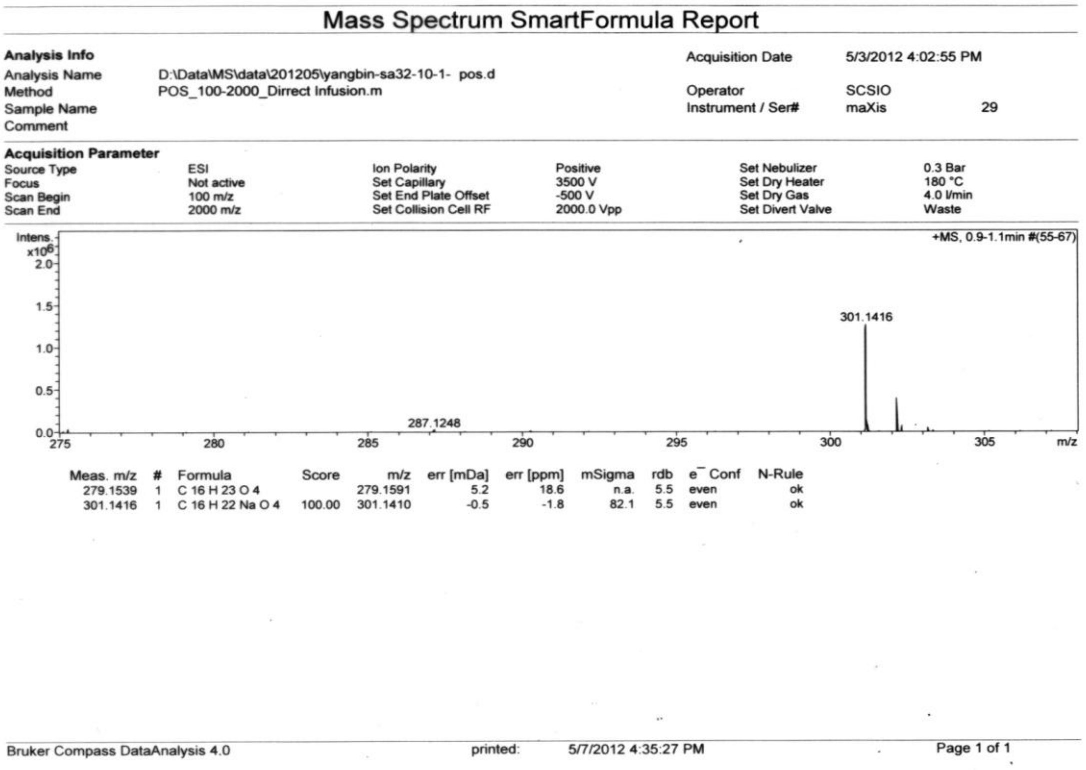

Figure S2. ^{1}H NMR Spectrum of sinularianin C (**3**) in CD_3OD (500 MHz).

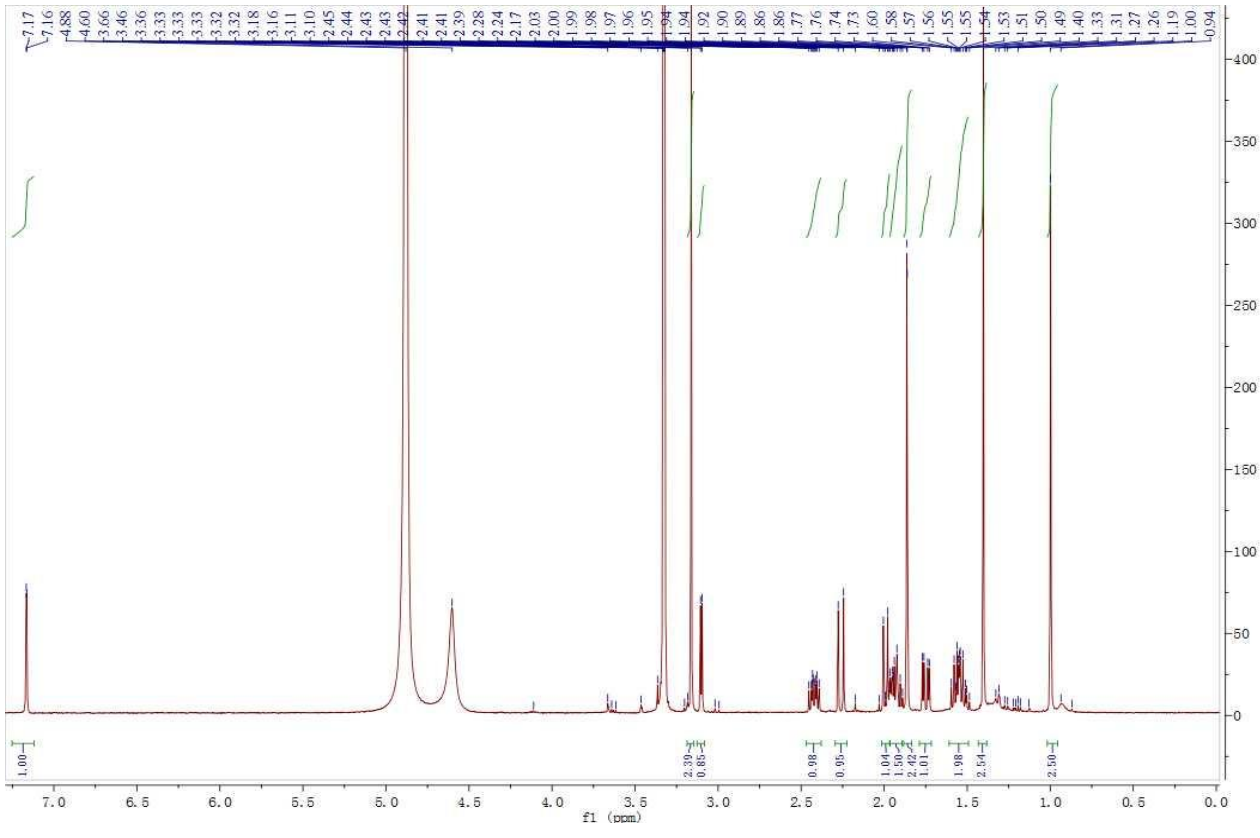

Figure S3. ^{13}C NMR Spectrum of sinularianin C (**3**) in CD_3OD (125 MHz).

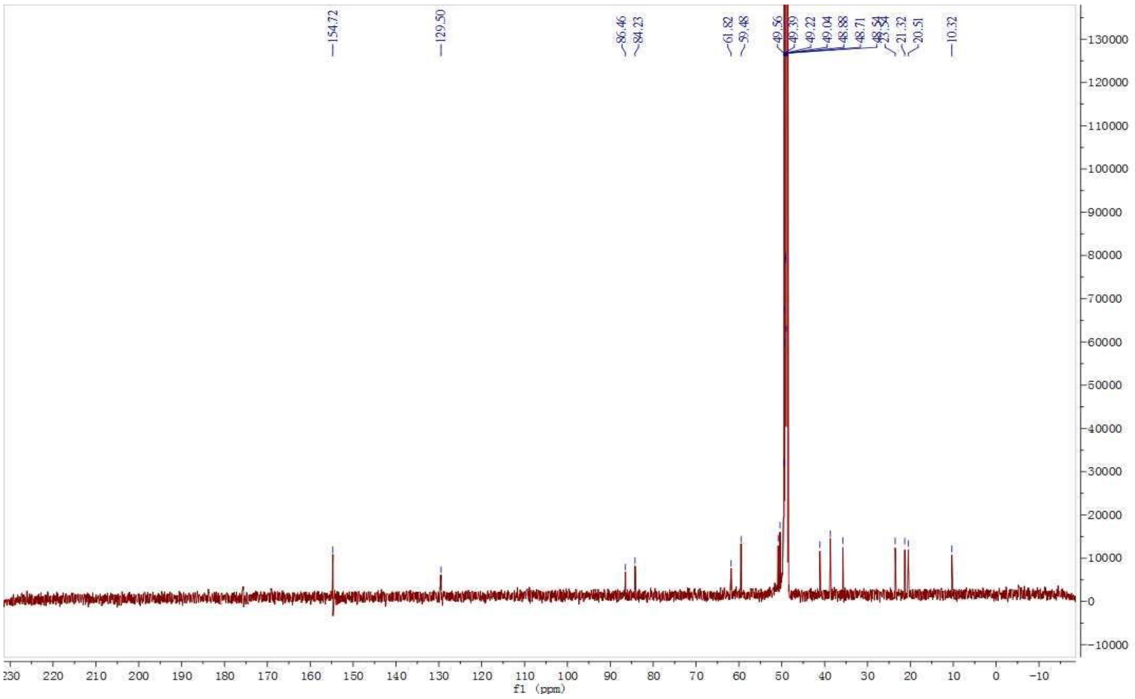

Figure S4. HMQC Spectrum of sinularianin C (**3**) in CD_3OD (500 MHz).

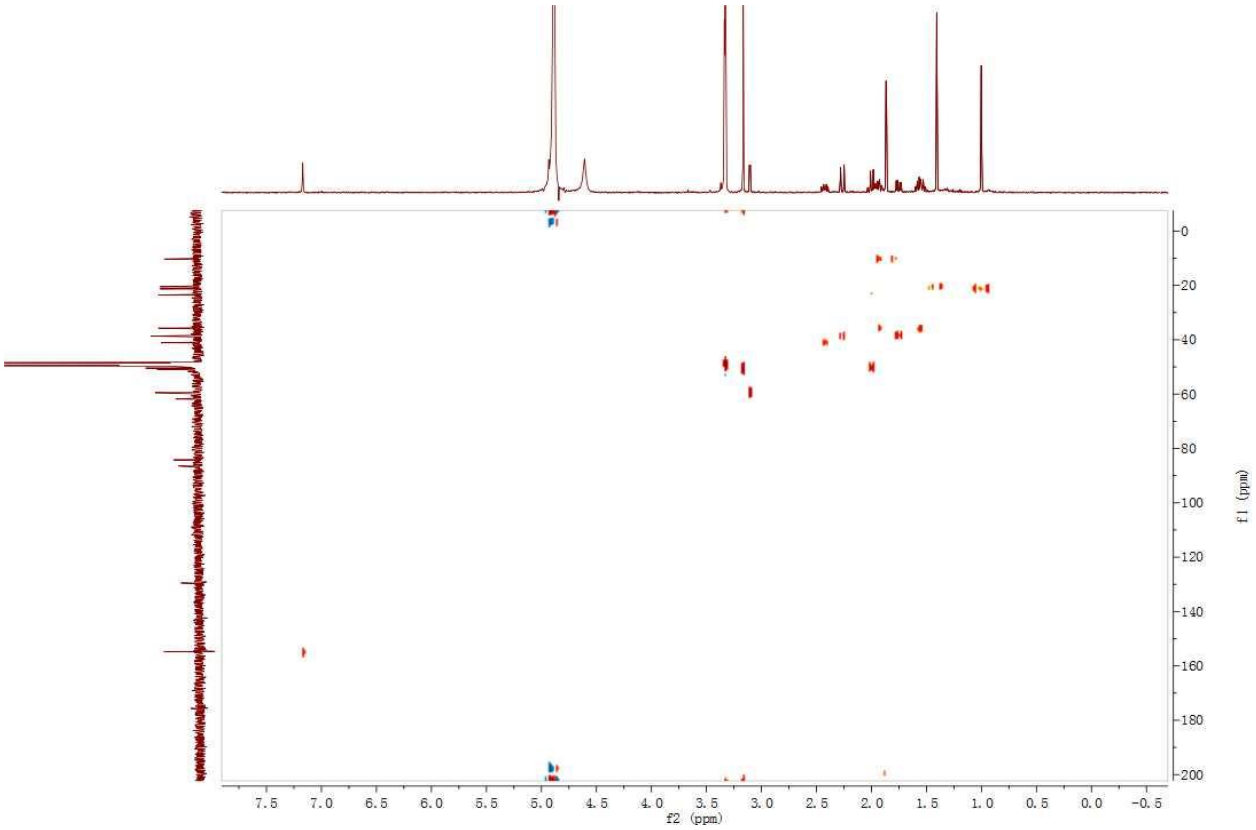

Figure S5. HMBC Spectrum of sinularianin C (**3**) in CD_3OD (500 MHz).

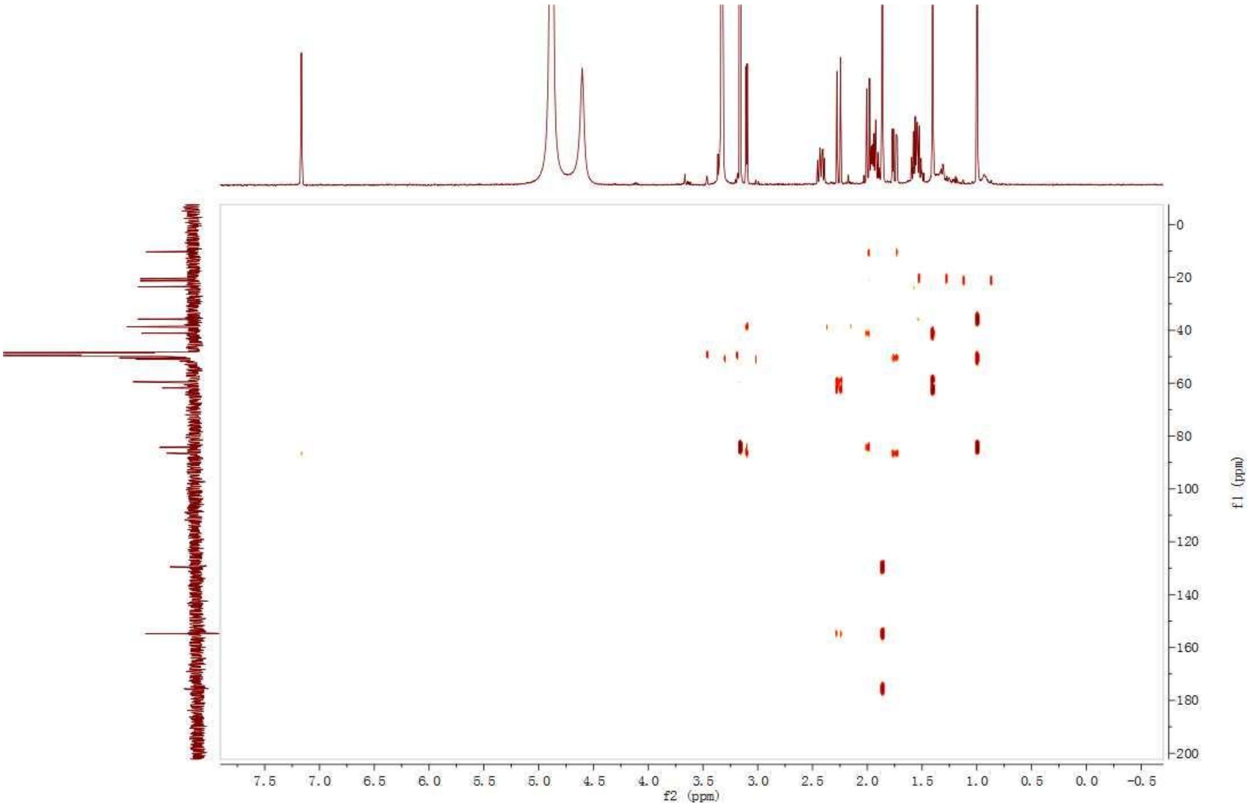

Figure S6. NOESY Spectrum of sinularianin C (**3**) in CD_3OD (500 MHz).

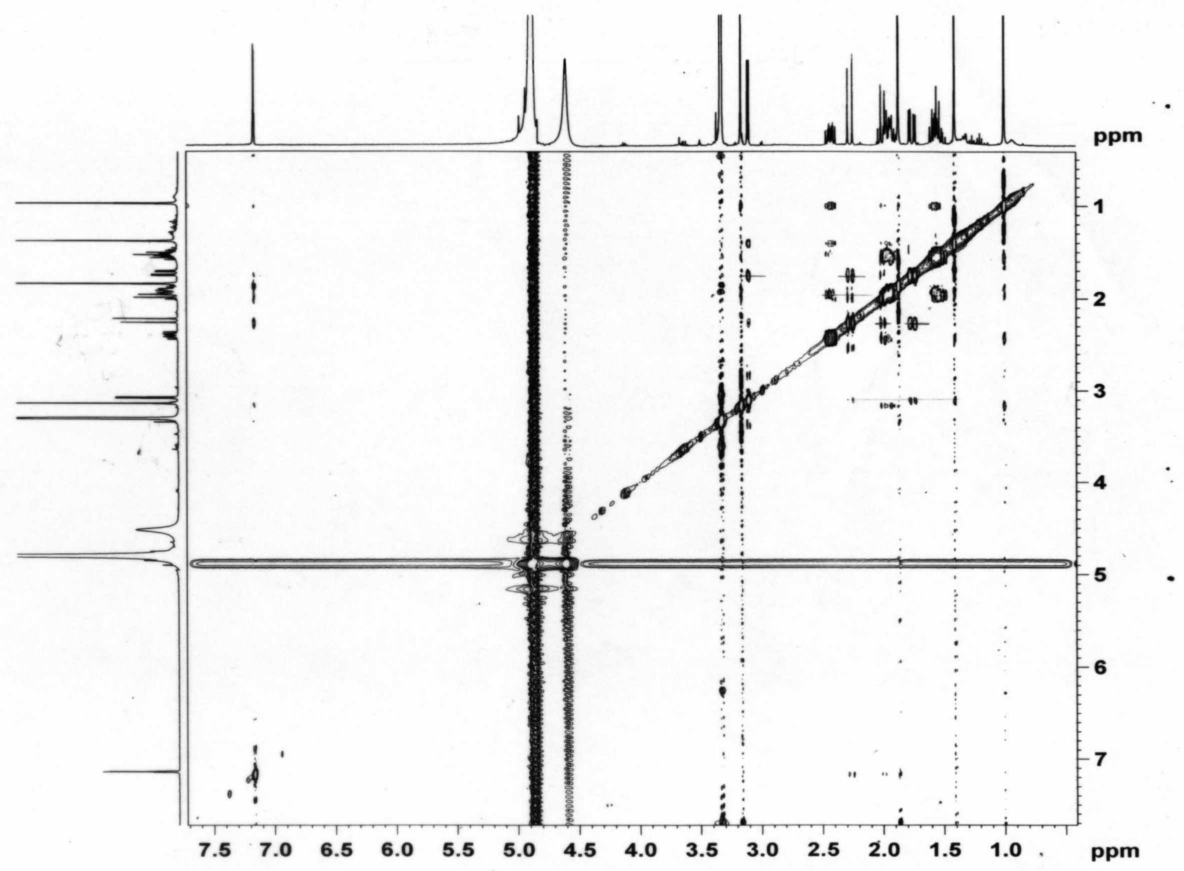

Figure S7. ESI-MS Spectrum of sinularianin D (**4**).

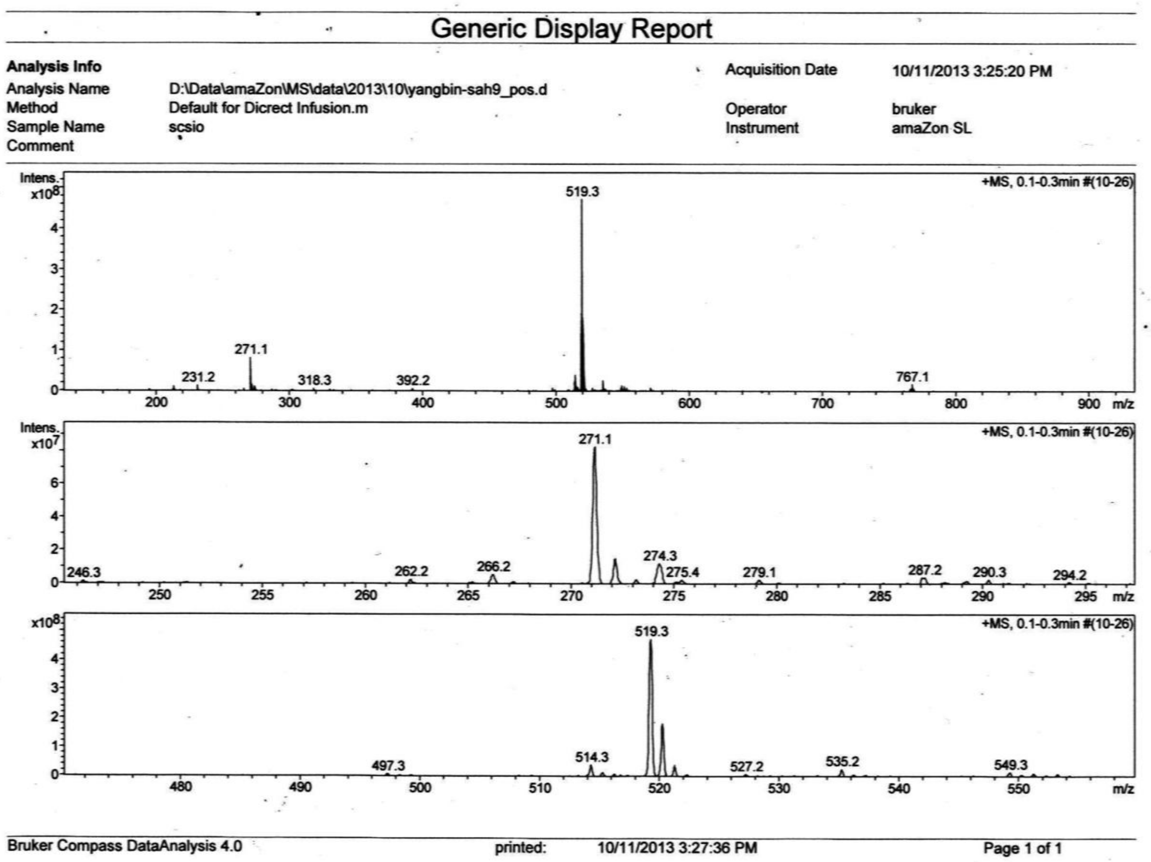

Figure S8. ^{1}H NMR Spectrum of sinularianin D (**4**) in $CDCl_3$ (500 MHz).

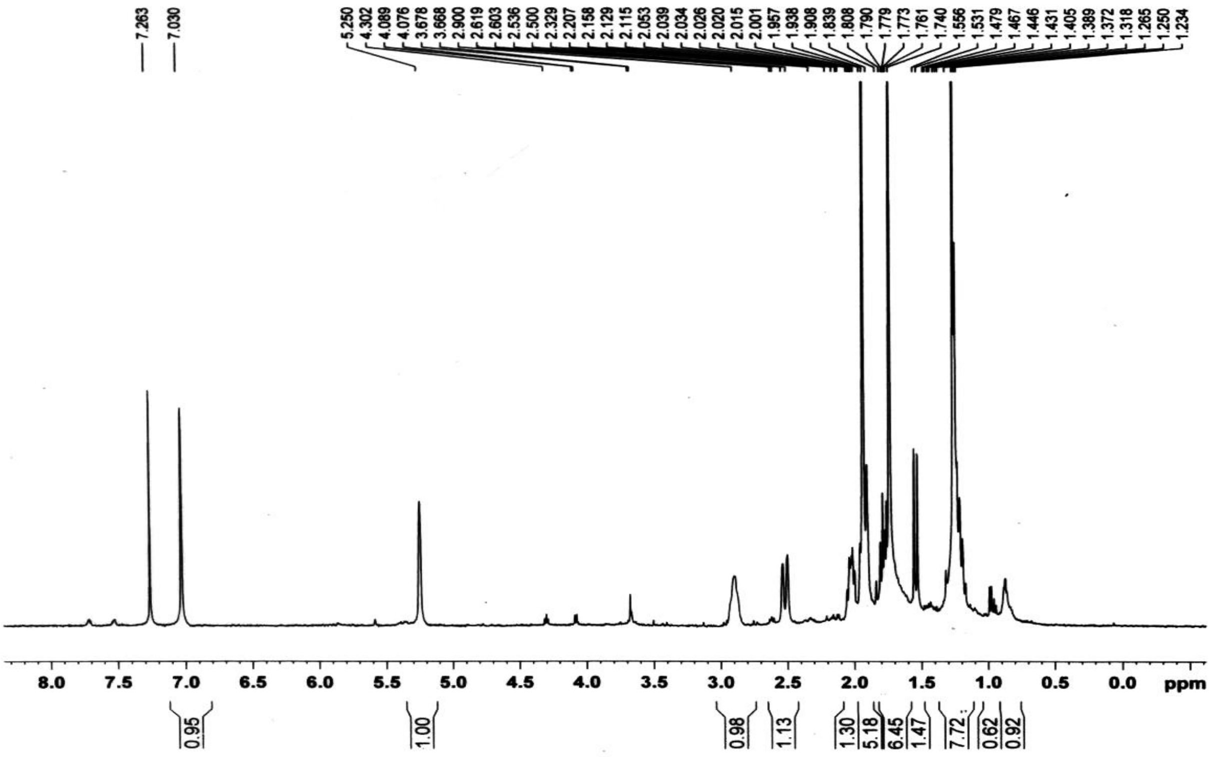

Figure S9. ^{13}C NMR Spectrum of sinularianin D (**4**) in $CDCl_3$ (125 MHz).

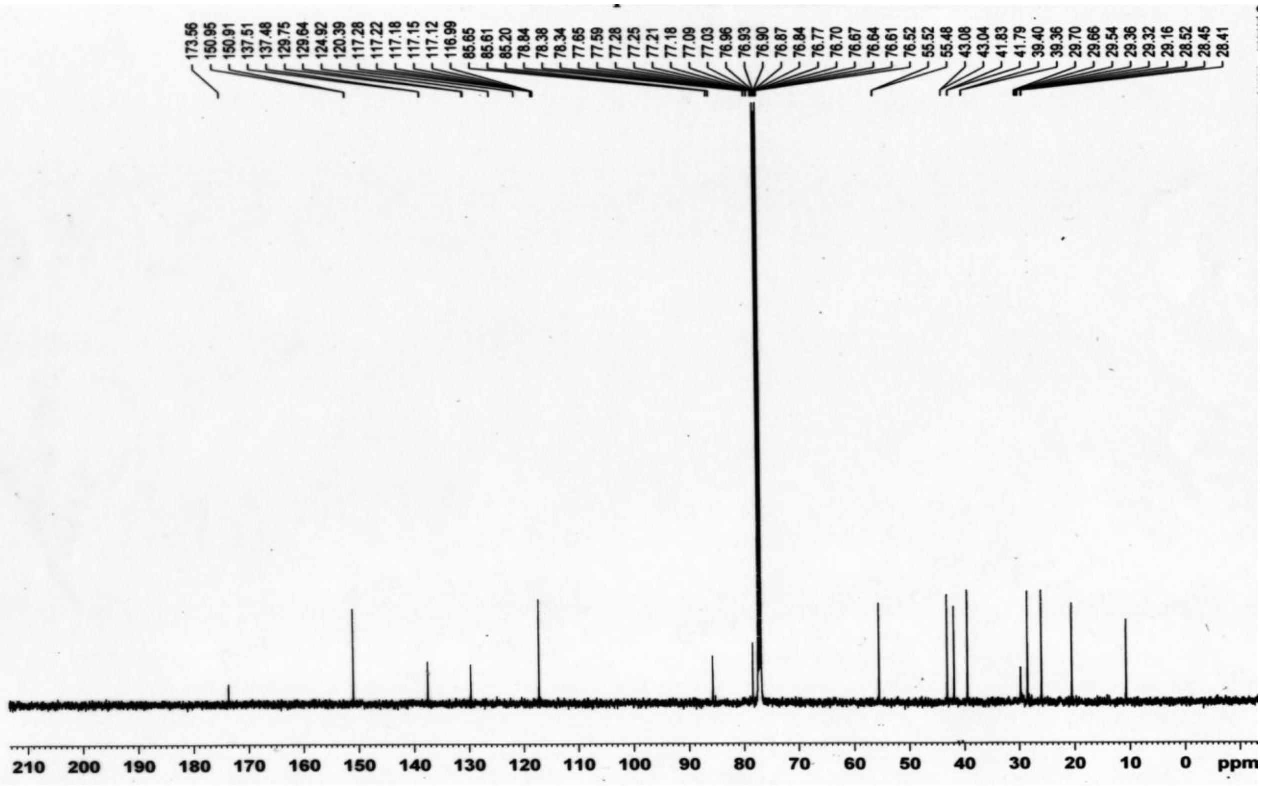

Figure S10. NOESY Spectrum of sinularianin D (**4**) in $CDCl_3$ (500 MHz).

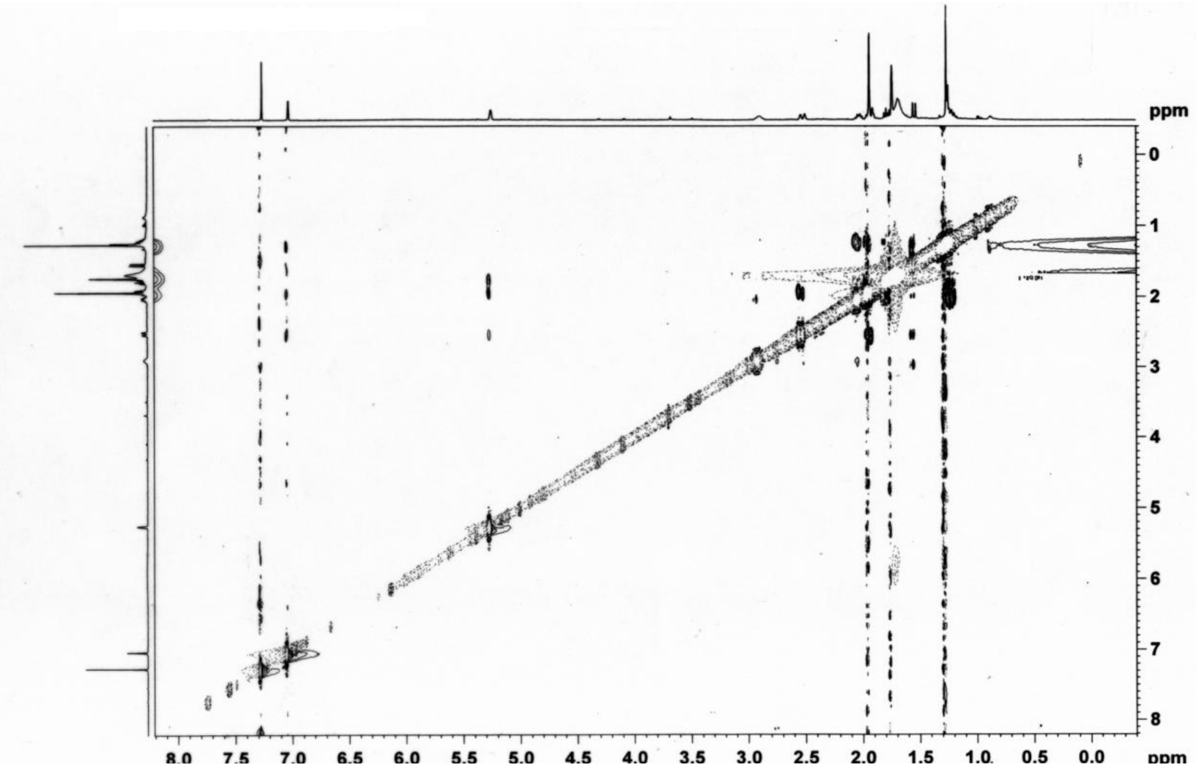

Figure S11. HR-ESI-MS Spectrum of sinularianin E (**5**).

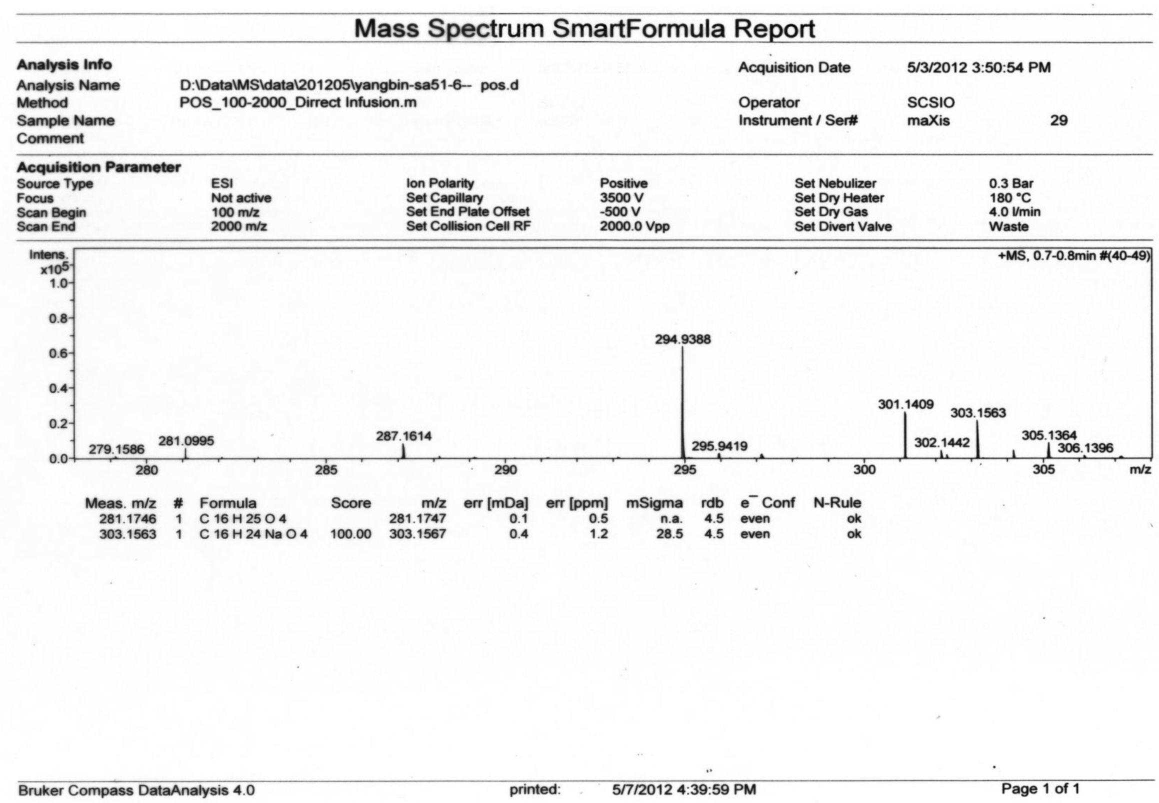

Figure S12. ^{1}H NMR Spectrum of sinularianin E (**5**) in $CDCl_3$ (500 MHz).

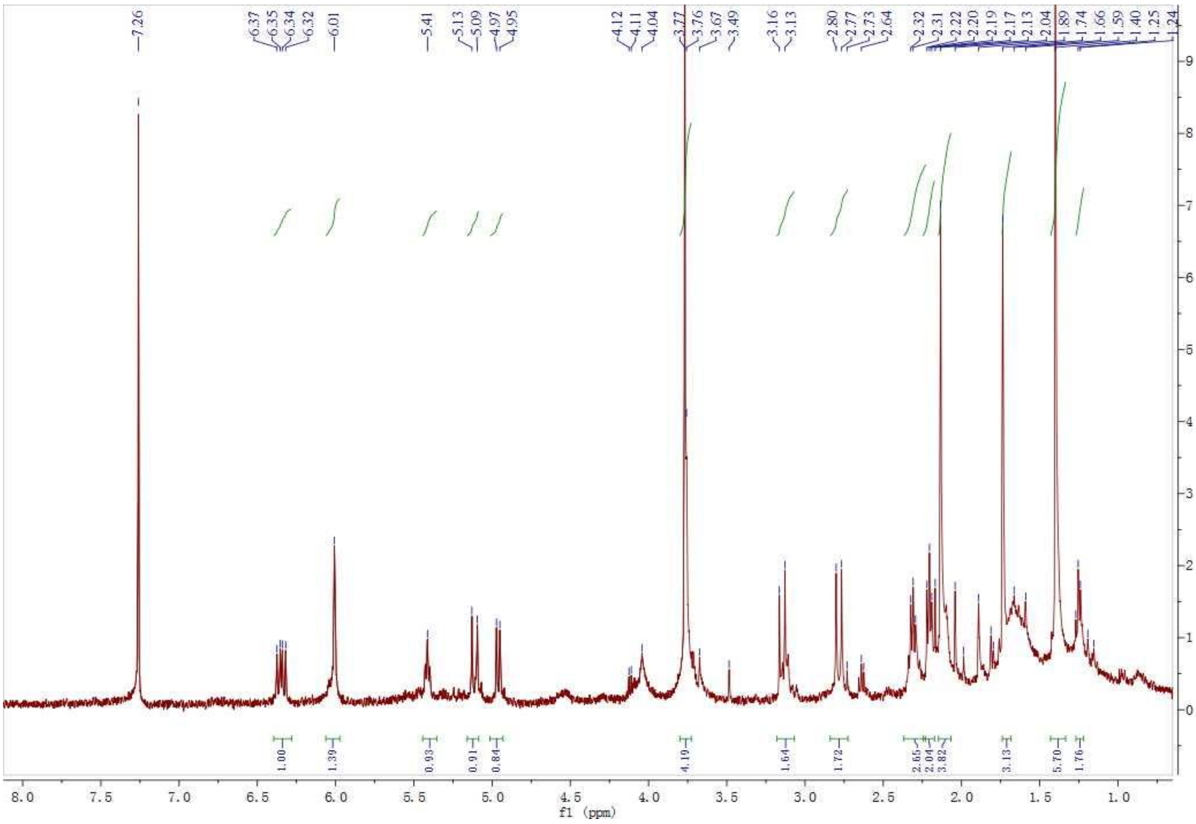

Figure S13. ^{13}C NMR Spectrum of sinularianin E (**5**) in $CDCl_3$ (125 MHz).

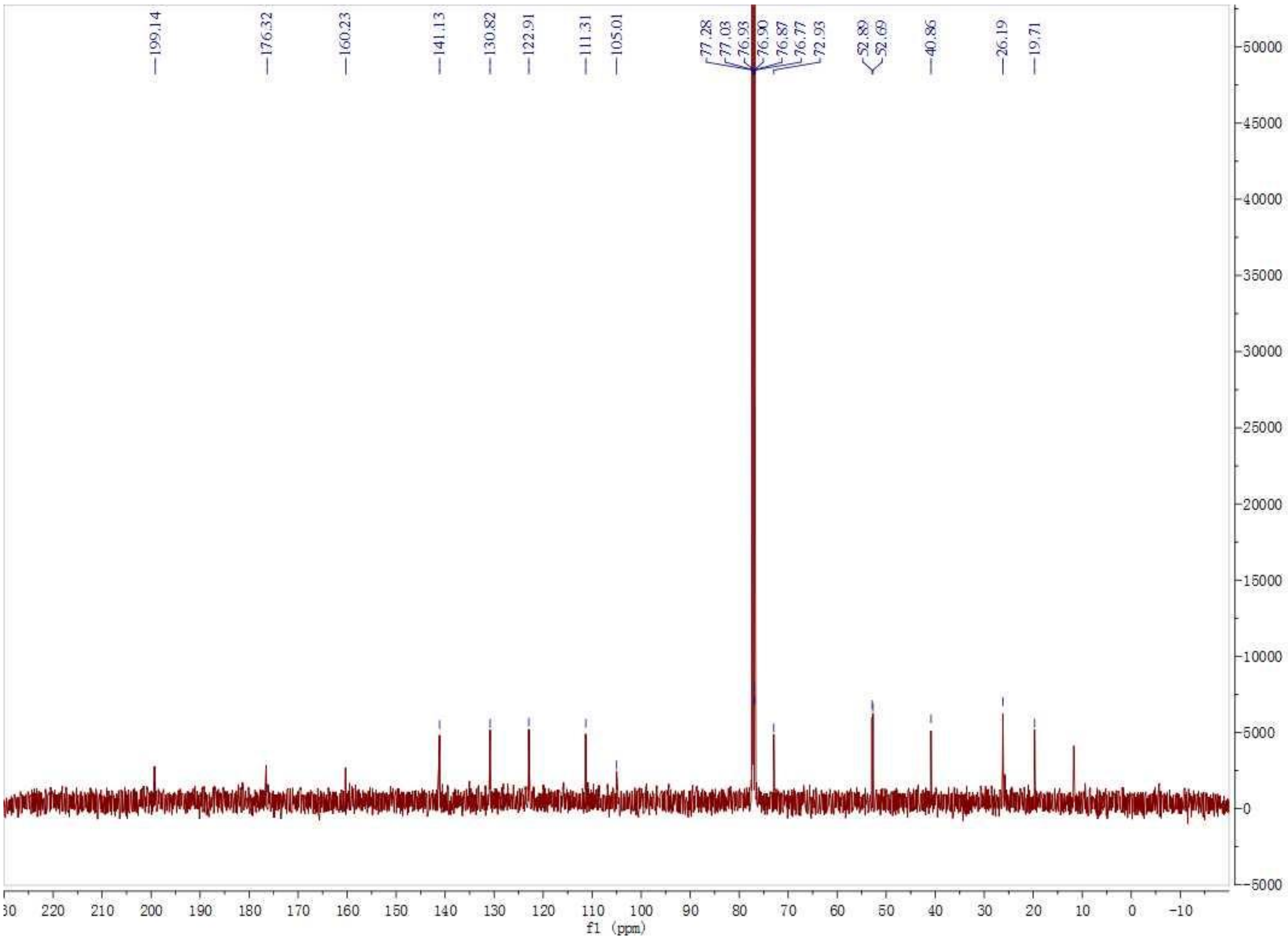

Figure S14. HMQC Spectrum of sinularianin E (**5**) in $CDCl_3$ (500 MHz).

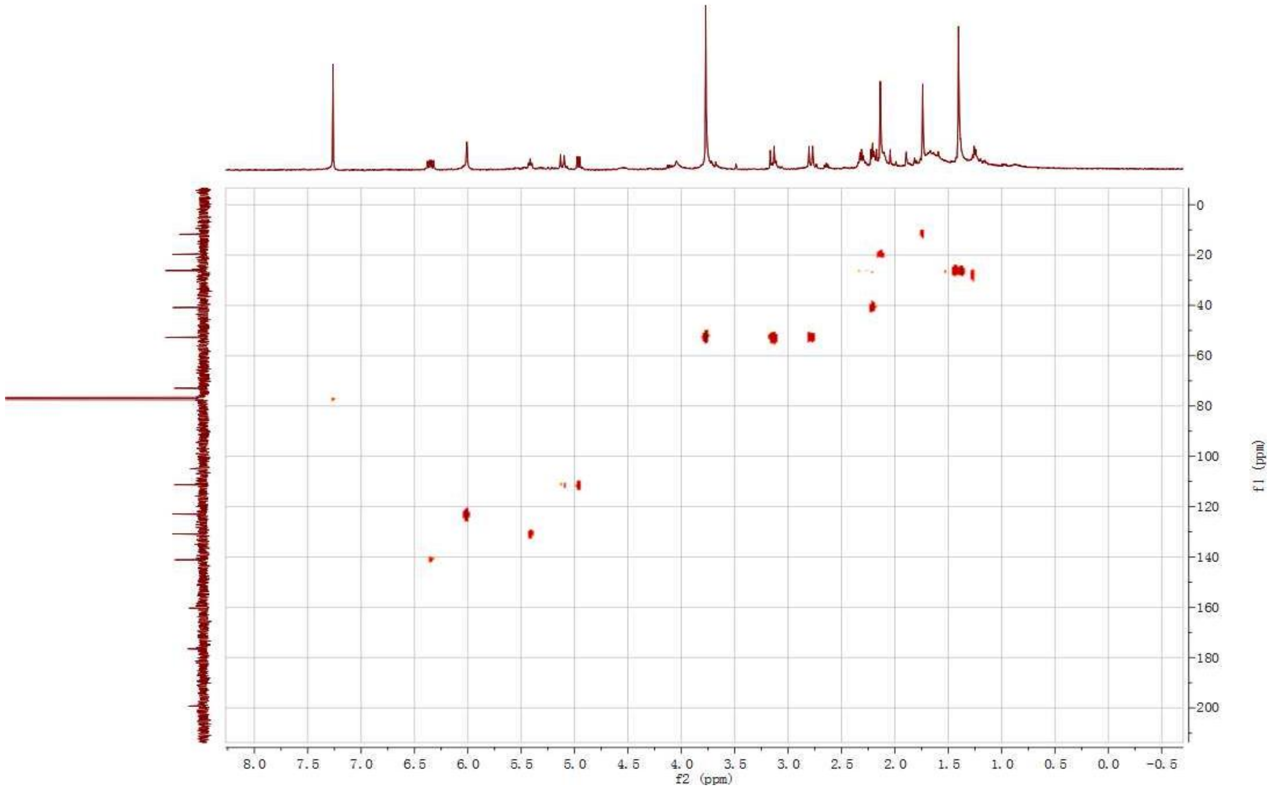

Figure S15. HMBC Spectrum of sinularianin E (**5**) in $CDCl_3$ (500 MHz).

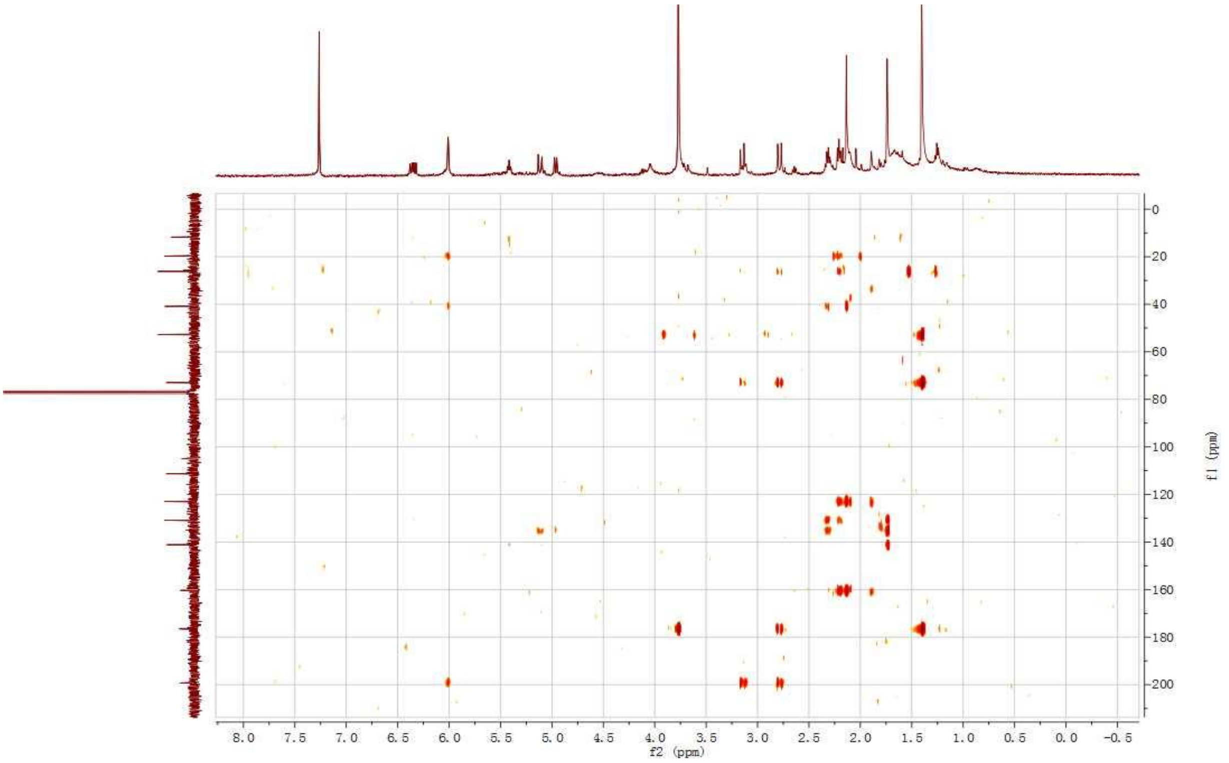

Figure S16. HR-ESI-MS Spectrum of sinularianin F (**6**).

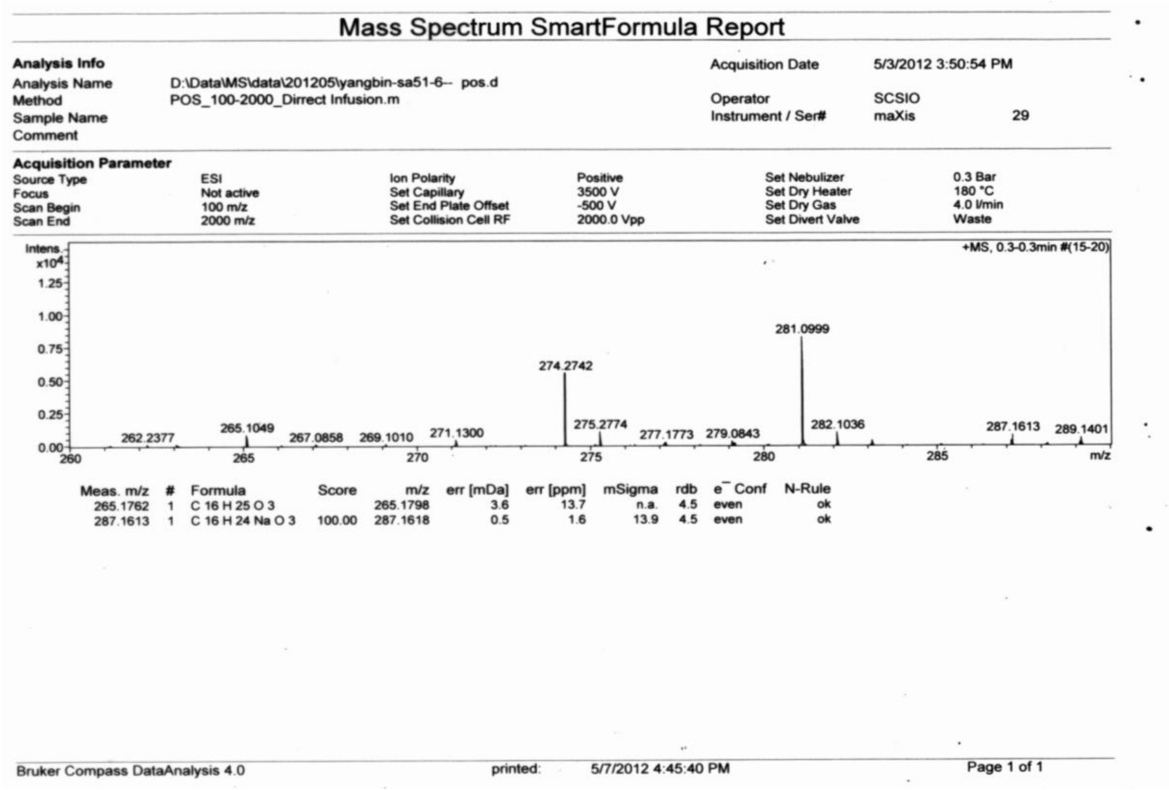

Mass Spectrum SmartFormula Report

**Analysis Info**		Acquisition Date	5/3/2012 3:50:54 PM
Analysis Name	D:\Data\MS\data\201205\yangbin-sa51-6-- pos.d		
Method	POS_100-2000_Dirrect Infusion.m	Operator	SCSIO
Sample Name		Instrument / Ser#	maXis 29
Comment			

**Acquisition Parameter**					
Source Type	ESI	Ion Polarity	Positive	Set Nebulizer	0.3 Bar
Focus	Not active	Set Capillary	3500 V	Set Dry Heater	180 °C
Scan Begin	100 m/z	Set End Plate Offset	-500 V	Set Dry Gas	4.0 l/min
Scan End	2000 m/z	Set Collision Cell RF	2000.0 Vpp	Set Divert Valve	Waste

Meas. m/z	#	Formula	Score	m/z	err [mDa]	err [ppm]	mSigma	rdb	e⁻ Conf	N-Rule
265.1762	1	C 16 H 25 O 3		265.1798	3.6	13.7	n.a.	4.5	even	ok
287.1613	1	C 16 H 24 Na O 3	100.00	287.1618	0.5	1.6	13.9	4.5	even	ok

Bruker Compass DataAnalysis 4.0 printed: 5/7/2012 4:45:40 PM Page 1 of 1

Figure S17. ^{1}H NMR Spectrum of sinularianin F (**6**) in $CDCl_3$ (500 MHz).

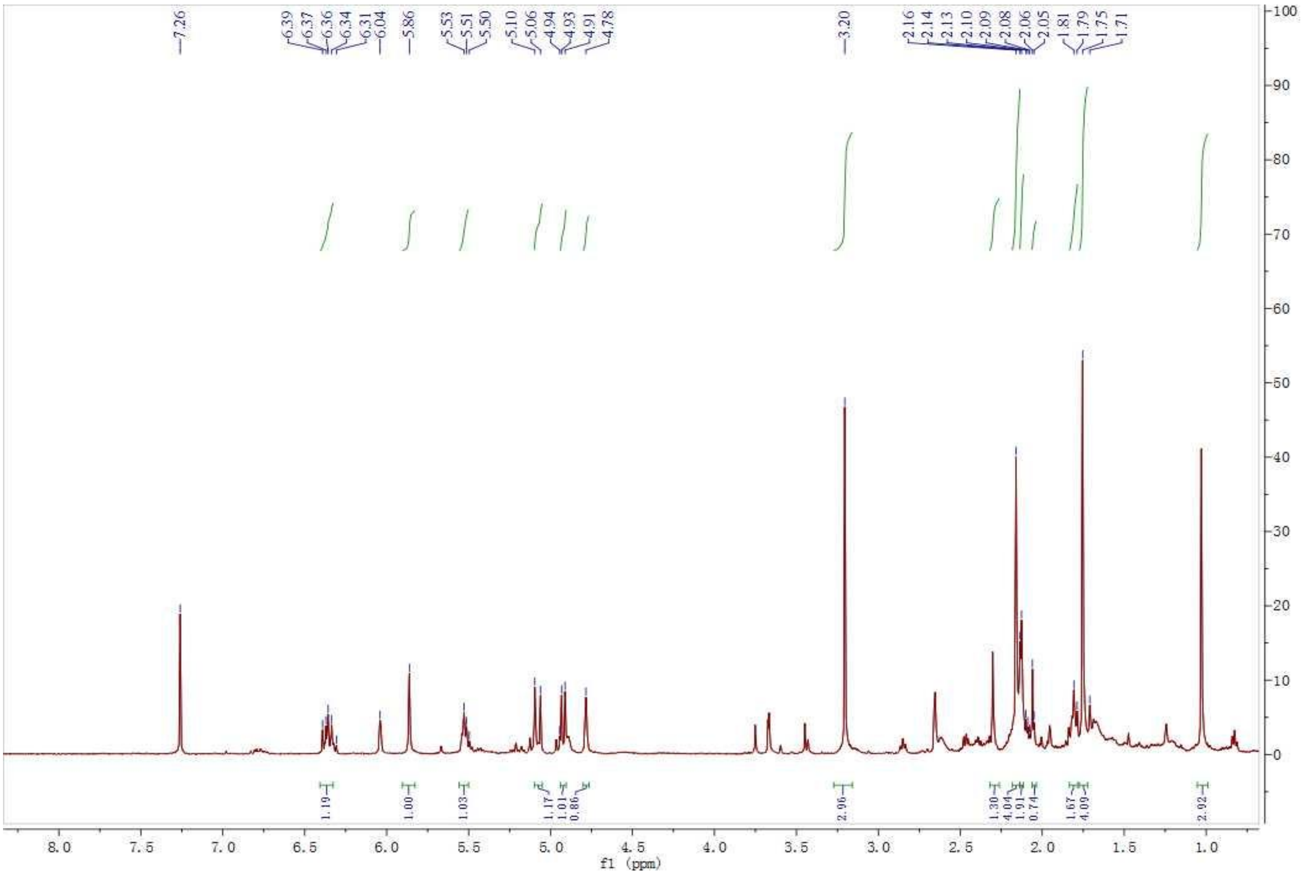

Figure S18. ^{13}C NMR Spectrum of sinularianin F (**6**) in $CDCl_3$ (125 MHz).

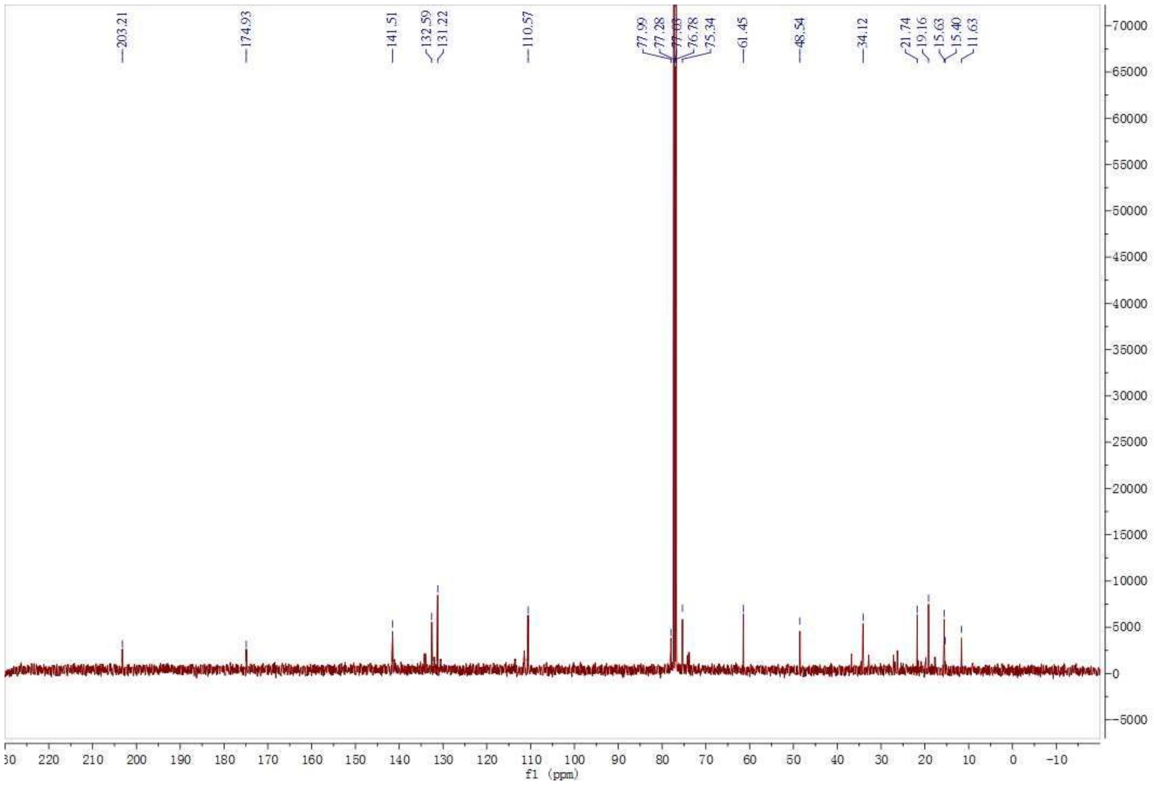

Figure S19. HMQC Spectrum of sinularianin F (**6**) in $CDCl_3$ (500 MHz).

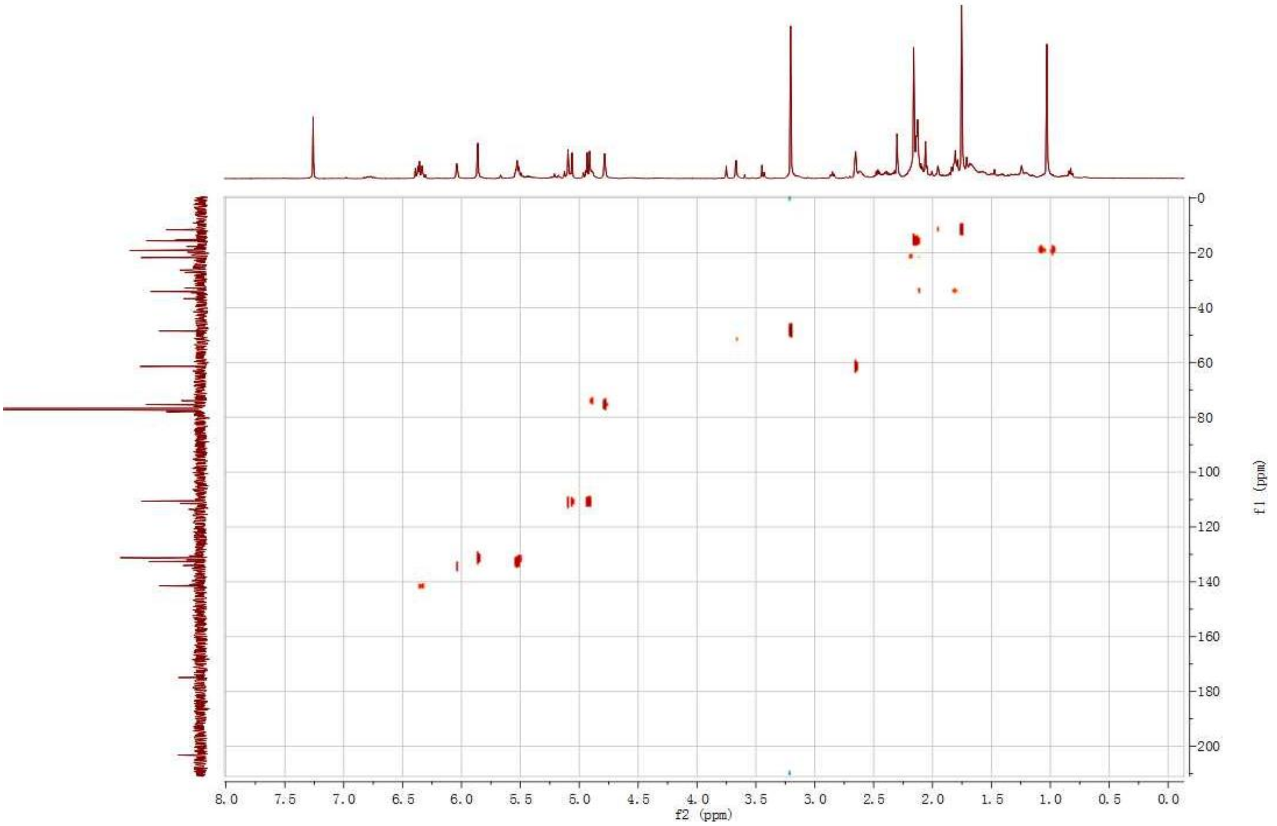

Figure S20. HMBC Spectrum of sinularianin F (**6**) in $CDCl_3$ (500 MHz).

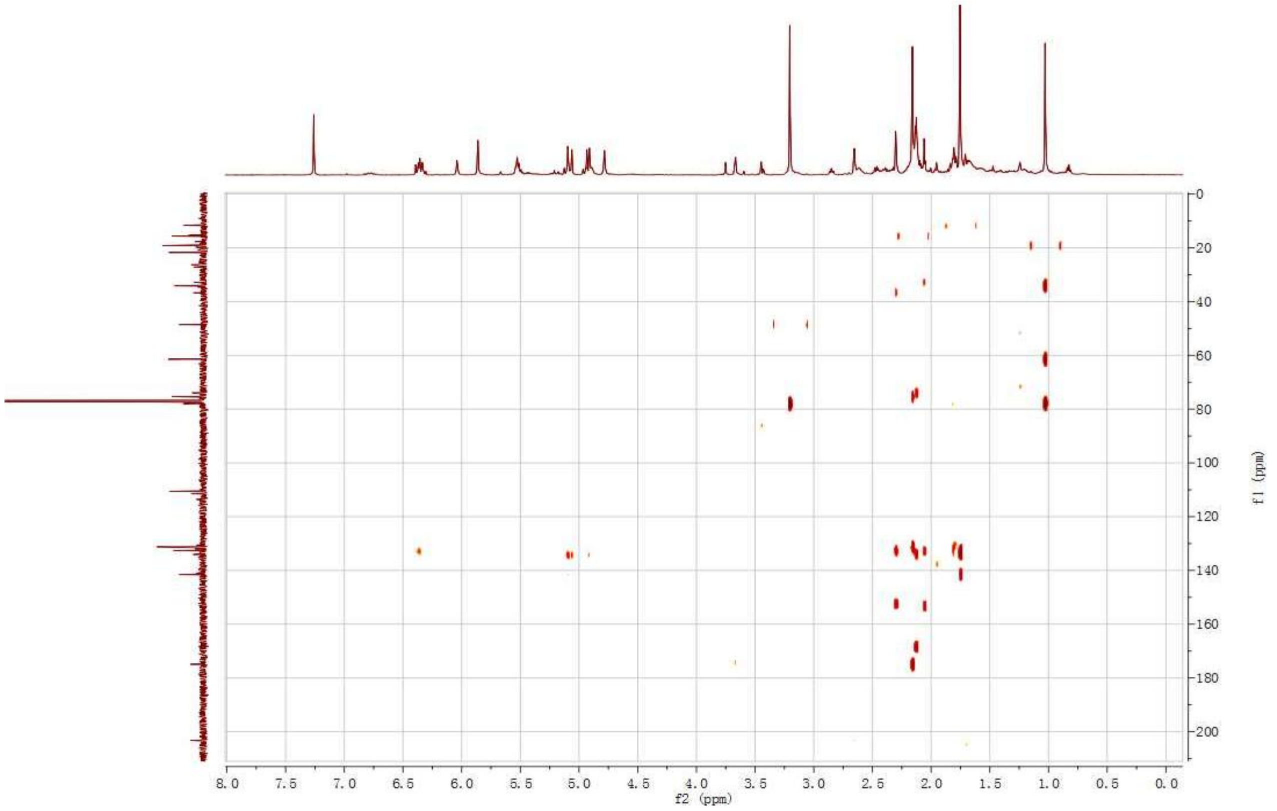

Figure S21. IR Spectra of sinularianin D (**4**).

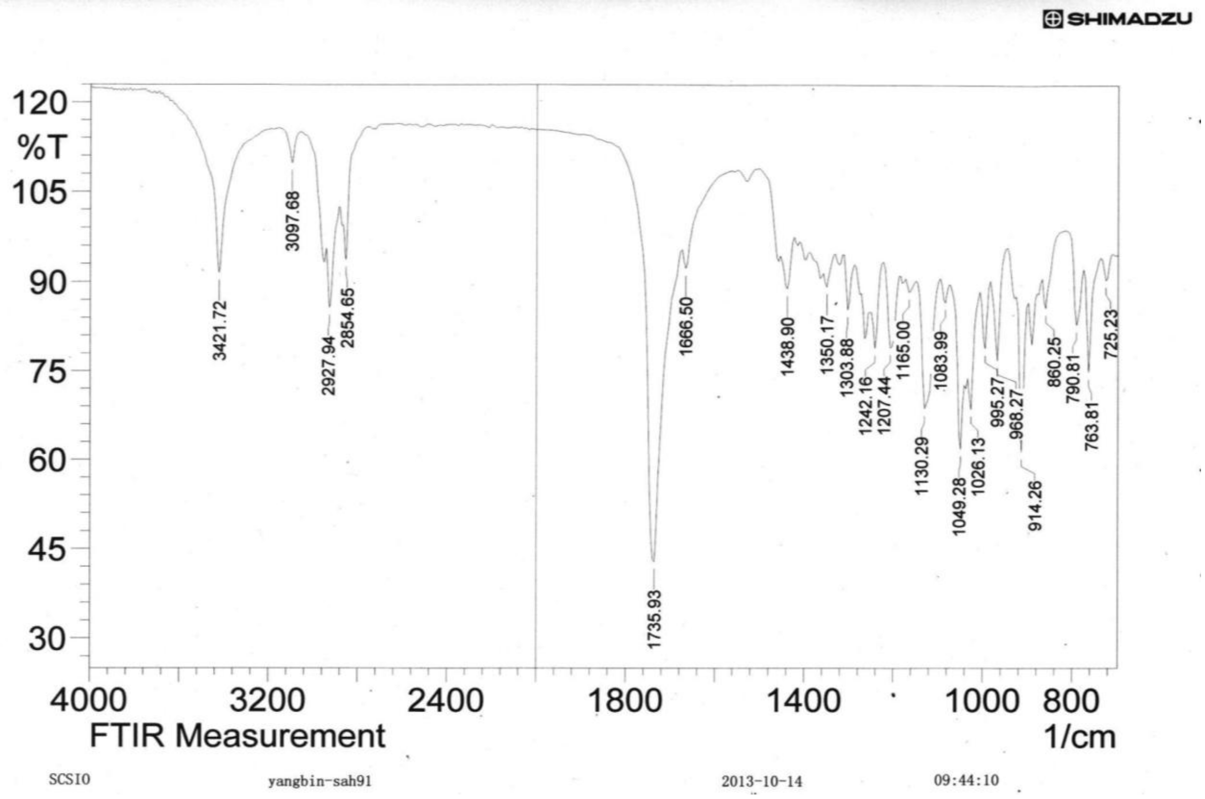

© 2013 by the authors; licensee MDPI, Basel, Switzerland. This article is an open access article distributed under the terms and conditions of the Creative Commons Attribution license (http://creativecommons.org/licenses/by/3.0/).
